# Appropriate Patient Selection in the Management of Common Bile Duct Stones: When Not to Do ERCP

**DOI:** 10.5402/2012/286365

**Published:** 2012-06-13

**Authors:** Palak Jitendrakumar Trivedi, Donald Tse, Ibrahim Al-Bakir, Horace D'Costa

**Affiliations:** ^1^Centre for Liver Research and NIHR Biomedical Research Unit, University of Birmingham, Edgbaston, Birmingham B15 2TT, UK; ^2^Department of Gastroenterology, Horton General Hospital, Oxford Road, Banbury OX16 9AL, UK; ^3^Department of Clinical Radiology, Horton General Hospital, Oxford Road, Banbury OX16 9AL, UK; ^4^Department of Surgery, Horton General Hospital, Oxford Road, Banbury OX16 9AL, UK

## Abstract

*Background*. Magnetic resonance cholangiopancreatography (MRCP) is noninvasive and accurate for diagnosing intra common bile duct stones (ICSs). However, given limited access, routine utilisation for investigating all patients with gallstone disease is neither practical nor cost-effective. Conversely, many individuals proceed directly to endoscopic retrograde cholangiopancreatography (ERCP), an invasive test with appreciable complications. *Aim*. Identify factors associated with ICS in order to improve risk-stratification for MRCP/ERCP. *Methods*. All patients having undergone cholecystectomy between November 2007 and October 2008 were reviewed. High-risk features for ICS were predefined, and their true presence confirmed by ERCP or intraoperative cholangiogram. Multivariate logistic regression was performed on candidate risk features. *Results*. Of 231 patients, 10.4% had ICS. Defining a high-risk group with “both” biochemical and ultrasound risk factors predicted ICS with 92% specificity and also bore strong association (OR 8.88). However, isolated hyperbilirubinaemia, ultrasound impression of CBD stones, and clinical risk factors did not (OR 1.10, 0.97, and 1.26). Normal liver biochemistry and normal ultrasound had a NPV of 99.5% for ICS. *Conclusions*. Ultrasound impression of CBD calculi without ductal dilatation is not predictive of ICS. Patients with normal liver biochemistry and normal CBD diameter on ultrasound are unlikely to have ICS and should not proceed to ERCP.

## 1. Introduction

Magnetic resonance cholangiopancreatography (MRCP) is well recognised to have high sensitivity and specificity (over 90%) for the detection of common bile duct (CBD) calculi [1–5]. However, a significant proportion of physicians allow patients with suspected intra-CBD stones (ICSs) to proceed directly to endoscopic retrograde cholangiopancreatography (ERCP), a procedure not without risk. ERCP has a high rate of complications, even in experienced hands. These may include post-ERCP pancreatitis (incidence rate 1.3–6.7%), haemorrhage (0.7–2%), and cholangitis (0.5–5%) [6–10]. The risk for ERCP-related complications is greatest in those with normal/marginal abnormalities in liver biochemistry and normal CBD diameter. In 2006, Cotton published a 10-year experience of being an expert witness in 59 cases that involved ERCP where malpractice was alleged [11]. The most common allegation (32 cases) was that ERCP (with/without sphincterotomy) was simply not indicated, with inadequate evidence for biliary (or pancreatic) pathology to necessitate said procedure. In over 30% of cases, there were no identifiable high risk factors which would arouse suspicion of ICS, yet these patients, perhaps unjustifiably, proceeded directly to ERCP, only to develop complications [11]. A follow-up report from 2010 identified an ongoing trend whereby no less than 20 cases of ERCP-related malpractice had taken place over the preceding 4 years, 45% of which related to patients having no high risk biochemical or transabdominal ultrasound (TAUS) features which supported the presence of ICS [12]. The American Society for Gastrointestinal Endoscopy guidelines [13, 14], the National Institutes of Health (NIH) State of the Science conference on ERCP [15], and the Birtish Society of Gastroenterology [16] state clearly that ERCP should be avoided if at all possible in such cases, with emphasis on noninvasive imaging, specifically MRCP as the diagnostic modalities of choice for confirming the presence of ICS, whilst reserving ERCP in cases where there is confidence an intervention will be required. Although MRCP is an accurate and noninvasive tool for the diagnosis of CBD calculi, access is still limited in many hospitals. Moreover, investigating all patients with gallstone disease for suspected ICS with MRCP would not be practical or cost-effective. Therefore, some form of patient selection or risk stratification based on the likelihood of CBD stone disease is required. Clinical features at presentation (jaundice, pancreatitis, ascending cholangitis), biochemical markers (elevated bilirubin, ALT/AST, and/or ALP), and findings on TAUS (dilated CBD, impression of CBD calculi) have been previously investigated for the ability to predict the risk of ICS. While findings such as a dilated CBD on TAUS or jaundice at presentation may increase the probability of CBD calculi, they suffer from low individual specificity [17]. Previous studies have demonstrated that models based on a combination of a patient's age, biochemical, and ultrasound findings can be developed to predict the presence of CBD stones [18–20] and therefore the need for further investigation or intervention. This has formed the basis of international guidelines [13, 14, 16]. 

The aims of this study were to identify factors which negatively predict the presence of ICS thus preclude the need for ERCP and devise a practicable algorithm for risk stratification in selecting patients with confirmed gallstone disease for MRCP versus proceeding directly to ERCP or cholecystectomy.

## 2. Study Design and Methods

### 2.1. Study Population

This is a single-centre study of all patients who underwent cholecystectomy in a North Oxfordshire (UK) hospital between November 2007 and October 2008. Patients were identified from operating theatre records, intraoperative cholangiogram (IOC) reports, ERCP reports, and in-patient case notes. All individuals who underwent cholecystectomy laparoscopic or open conversion were included. This retrospective study received institutional board approval, and informed consent was waived.

### 2.2. Risk Factors

Patients' medical records, including biochemical and radiological databases, were analysed for the presence of risk factors indicative of CBD calculi. For the clinical presentation, a history of jaundice, pancreatitis, or cholangitis was selected as “high-risk” factors based on findings from previous studies and meta-analysis [17, 18], while the absence of all three was classified as low risk. Perioperative results of liver associated enzymes (ALP and ALT) and bilirubin were reviewed to assess biochemical risk factors, and the highest levels of each were recorded from time of first presentation to 30 days after cholecystectomy. Any biochemical test value above our laboratory's local upper limit of normal (ALT, 45 IU; ALP, 330 IU; bilirubin 17 *μ*mol/L) was classified as “high-risk.” For the presence or absence of ultrasound indicators, the presence of a CBD diameter greater than 7 mm and/or an impression of ICS on TAUS defined TAUS high risk.

### 2.3. Verification of CBD Stone Disease

The presence or absence of CBD stones was confirmed by followup of the medical notes, review of ERCP reports, and radiological databases, and when present, results of an intraoperative cholangiogram (IOC). Hospital admission records and further imaging performed in the 30 days following cholecystectomy were also analysed for evidence of ICS presenting postoperatively. 

### 2.4. Statistical Analysis

Data was analysed using PASW Statistics version 18.0 (SPSS, Chicago, IL, USA). Binomial logistic regression analysis for the prediction of CBD stones was performed using candidate factors of clinical risk, biochemical risk, and ultrasound risk. Further binomial logistic regression analyses were performed using the level of each individual biochemical test as candidate factors in one analysis and a dilated CBD diameter (>7 mm) and/or TAUS evidence of ICS in the other. Odds ratios (ORs), sensitivity, and specificity of each individual risk factor were subsequently calculated.

## 3. Results

### 3.1. Patient Characteristics

The patient cohort included 231 patients (182 female) with a mean age of 50 years (18–77). 10.4% (*n* = 24) were diagnosed with CBD stones preoperatively (*n* = 17), intraoperatively (*n* = 4), or postoperatively (*n* = 3). 

### 3.2. The Presence of Elevated ALT or ALP, but Not Bilirubin in Isolation, Is Associated with the Presence of CBD Calculi

A high biochemical risk, meaning high levels in one or more of the three tests of liver function, was associated with a significant odds ratio (OR) of 23.9 for the presence of CBD stones (Table 1). The three liver function tests studied (ALT, ALP, and bilirubin) were analysed as separate risk factors in a second logistic regression using only these factors. As shown in Table 2, both a high ALT and a high ALP were independently associated with a significantly high odds ratios of 13.7 and 7.2, respectively. The OR for a raised bilirubin in isolation, however, was only mildly raised at 1.10 and did not reach statistical significance. Abnormal liver biochemistry predicted the presence of CBD stones with 96% sensitivity and 59% specificity. Only 1 patient (0.85%) with normal ALT and normal ALP was eventually diagnosed as having a CBD stone (postoperatively).

### 3.3. Dilated CBD Diameter (>7 mm) Is Associated with the Presence of ICS

High TAUS risk was associated with a significant OR of 3.03 (Tables 1 and 3). When analysing these findings individually, a dilated CBD in isolation had a greater association with the eventual presence of CBD stones (OR 6.53). Only 13/197 (6%) of patients with a normal CBD diameter were subsequently diagnosed as having ICS. The impression of ICS on TAUS in the absence of a dilated CBD did not give rise to a statistically significant OR (0.97).

### 3.4. Clinical Risk

High clinical risk based on a history of jaundice, pancreatitis, or cholangitis demonstrated a slightly increased but nonsignificant OR of 1.26 (95% confidence interval 0.46–3.45). Neither patient age nor sex influenced the odds of CBD calculi being present (data not shown).

### 3.5. The Combination of Biochemical and TAUS Risk Factors Defines the Highest Risk Group for the Eventual Presence of CBD Calculi

We combined the biochemical and TAUS risk factors to define a group which included patients with *either* high biochemical risk *and/or* a high ultrasound risk, such that this group included all patients with one or more abnormalities in liver function tests *and/or* abnormalities on TAUS. This group contained 114 patients (49% of the total cohort) in whom 23 (20.1%) were subsequently confirmed to have CBD calculi. Using this “and/or” method yielded a sensitivity of 96% and specificity of 56% for detecting ICS disease (Table 1). Refining this combined risk group further, the presence of *both* abnormal TAUS *and *abnormal liver biochemistry yielded a higher specificity (92%), with an OR of 8.88 on binominal logistic regression (Table 1).

Out of 117 patients with normal liver function tests and normal CBD on ultrasound, only 1 patient (0.85%) had a CBD stone subsequently detected (identified postoperatively). The negative predictive value of a normal CBD diameter on TAUS *and* normal liver biochemistry in the perioperative cholecystectomy period was 99.5% for the eventual presence of ICS.

## 4. Discussion

Investigating individual liver function tests and the different CBD findings on ultrasound identified an increased ALT, increased ALP, or a CBD diameter greater than 7 mm as the most significant risk factors for ICS. We have also shown that patients with *both* abnormal liver function tests *and* abnormal TAUS CBD appearances (high-risk group) are more likely to have CBD calculi compared to those with normal test results. Selecting this group would include the vast majority of patients with CBD calculi. Thus, as this “combination” group bears the highest specificity, it may be reasonable for such patients to proceed directly to ERCP for biliary intervention thereby circumventing the need for MRCP. However, MRCP provides clear additional benefits for the endoscopist by delineating the ductal anatomy, which is often not well demonstrated by TAUS alone and therefore anticipated that, where available, MRCP may still be a useful intermediate step prior to ERCP. This decision should be made on a case-by-case basis.

In those patients in whom *neither* liver-associated enzymes *nor* CBD dilatation is present (low risk group), it is extremely unlikely that there are CBD calculi present. In this group, it is reasonable to proceed directly to cholecystectomy where indicated. The group in whom *either *ductal dilatation *or* abnormal biochemistry is present (intermediate-risk group) still have a 29-fold higher chance of having CBD calculi compared to those in the low-risk group. Although this predicts the presence of ICS with high sensitivity (96%), using this “*either*/*or*” approach still suffers from low specificity hence patients falling into this category are still advised to undergo MRCP prior to definitive biliary intervention due to the high false-positive rate. 

The low diagnostic accuracy of detecting ICS by visualising CBD stones on TAUS (in the presence of normal CBD diameter) can be attributed to a number of factors. Firstly, there isan acknowledged difficulty amongst radiologists that interrogate the biliary treein reliably identifying intraductal calculi in the extrahepatic biliary system. This is usually due to overlying bowel gasthat precludes identifying the calculus accurately despite manoeuvring the patient.In this situation, abdominal gas within a viscus dissipates the focussed ultrasound beam resulting in incoherent/nondiagnostic images beingproduced. Secondly, the calculus depending on its size may not necessarily cast an acoustic shadow,and their location near the ampulla can bevery difficult to detect on ultrasound. Conversely, the appearance of an acoustic shadow may be caused by refraction of the ultrasound beam or mucosal junctional folds which may falsely simulate the appearance of ICS. Finally, it is also important to note that, when intraductal calculi are detected, the delay between the ultrasound study and ERCP/IOCmay besubstantial, resulting in the passage of the calculus before the latter invasive studies are performed. It is worth noting that MRCP has a higher sensitivity particularly in detecting small CBD calculi, particularly if the fluid in the duodenum is suppressed (e.g., with pineapple juice within the duodenum). This is due to the magnesium content within the juice that suppresses the fluid signal.

An alternative diagnostic modality for the evaluation of ICS is endoscopic ultrasound (EUS), which in expert hands provides excellent images of the common bile duct. Several studies have compared the diagnostic accuracy of EUS to ERCP in patients with moderate to high risk of having ICS. Taken collectively, the sensitivity of ERCP for ICS in these studies ranges from 79 to 100% compared to 84–100% for EUS, and the specificity from 87 to 100% for ERCP compared to 96–100% for EUS [16]. Neither test is consistently demonstrated to be superior when results of individual studies are examined. However, EUS is not yet widely available and still requires the patient to undergo endoscopy without adequate visualisation of the intrahepatic ducts.

**Figure 1:**
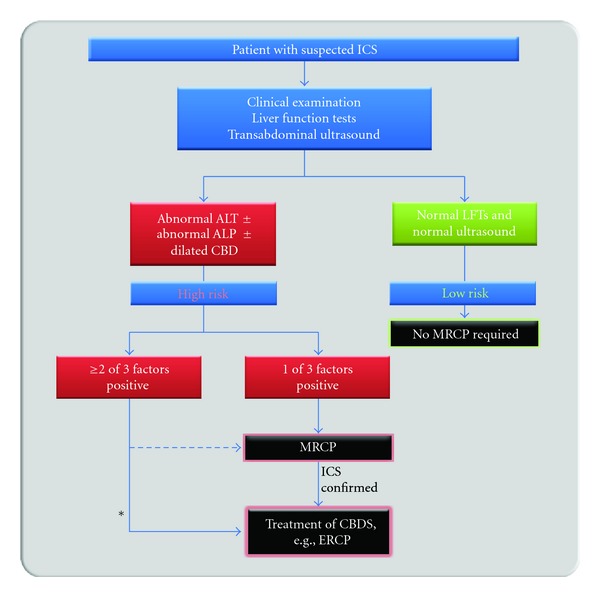
Patient selection for MRCP and ERCP based on risk stratification. *For those individuals in whom CBD stones are suspected, the combination of abnormal liver function tests and a dilated CBD diameter (>7 mm) identify the vast majority of patients who have true, intraductal calculi. In this group, it is not unreasonable to proceed directly to ERCP. However, in those individuals who have only 1 of the 2 abnormalities present, the risk is intermediate, and hence MRCP as a noninvasive modality is needed. Those patients in whom neither liver function tests nor TAUS features are abnormal are at low risk of having intraductal stones and may proceed directly to cholecystectomy.

The results of our study lead us to propose a pathway to select those at intermediate to high risk for further investigation with MRCP. If this pathway was applied to our sample group, a total of 114 patients (49%) would have undergone MRCP with 23 out of 24 patients having CBD stones being correctly identified. Compared to 84 patients (36%) who actually received MRCP in our cohort, this is a significant but still practical increase in demand. While MRCP has benefits in being a noninvasive and accurate diagnostic test, it can however be viewed as an unnecessary step in the management of patients who will almost definitely have a CBD stone and require therapeutic ductal intervention. Hence, as already discussed, it may be justified to proceed directly to ERCP should the aforementioned high-risk indicators be present. This view is supported in the British Society of Gastroenterology (BSG) guidelines as a reasonable and cost-effective strategy [16], and, given variations in the accessibility to MRCP, this would also be relevant to many centres. Therefore, in our proposed pathway (Figure 1), we have suggested that a subgroup of patients in whom *both* liver-associated enzymes (either ALT or ALP) *and* CBD diameter are abnormal (high specificity) could be considered for direct referral to ERCP. This may be especially useful in circumstances where difficult access to MRCP means that there would be undue delays for the diagnosis to be confirmed and definitive treatment offered to a high-risk patient.

While other studies with larger sample sizes have developed similar predictive models based on patient age, clinical presentation, biochemistry, and ultrasound findings, they were developed such that the next step in the management pathway was either ERCP or IOC, both invasive procedures with associated risks. Because of the risks associated with ERCP or IOC, these models required a high specificity and high positive predictive value in which those selected would have a high likelihood of requiring therapy [19]. Although only 20% of our intermediate-risk group and 38% of our high-risk group eventually had CBD stone disease, all-but-one patients with ICS would have been included in these categories. Thus, we have identified an accurate method of detecting those patients at low risk of needing invasive biliary intervention which may reduce the development of ERCP-related complications.

Limitations to our study include the relatively small sample size from a single centre, which led to wide confidence intervals for our calculations of odds ratios. However, we hope that this study would provide a basis for larger scale studies to be carried out. Secondly, it is likely, but not presumed in this study, that there is an association among abnormal individual liver function tests and CBD abnormalities on ultrasound; therefore, the calculated odds ratios in the multivariate logistic regression analyses would not be simply multiplied in the case where more than one risk factor is positive. Thirdly, there is a variable gap of time between liver function tests, TAUS, and verification of CBD stone disease. This raises the probability that CBD calculi could have developed or resolved in this time period. Finally, a useful measure would be serial monitoring of liver associated enzymes and bilirubin over time rather than at one time point as in our study. This would enable us to trend whether normalisation in liver function tests equates to the successful ductal passage of calculi. We believe that, while this would influence the accurate predictive value of biochemistry and ultrasound, this more closely reflects what happens in routine clinical practice which is important given access pressures for investigations and procedures such as MRCP or ERCP.

In conclusion, patients with suspected ICS can be risk stratified and appropriately selected for further investigation by MRCP or proceed straight to therapeutic ERCP. Individuals with *either *abnormal liver biochemistry *or* CBD appearances on TAUS may need further assessment with MRCP; however, patients in whom *both* investigations are abnormal may proceed directly to ERCP as MRCP may delay therapeutic intervention or be of limited availability. Moreover, those with normal liver biochemistry and normal appearances of the CBD on TAUS are exceedingly unlikely to have CBD stone disease and thus ERCP is not warranted in this setting. Adopting this approach may avoid unnecessary complications. We have proposed an algorithm for patient selection that is practicable and useful at our centre.

## Figures and Tables

**Table 1:** Clinical, biochemical, and ultrasound risk features for the presence of CBD stones.

Risk factor	Odds ratio	95% C.I.	Sensitivity	Specificity
Clinical^1^	1.26	0.46–3.45	42%	86%
Biochemical^2^	23.9	3.0–188	96%	59%
Ultrasound^3^	3.03	1.12–8.19	46%	88%
Biochemical *and/or* ultrasound	29.3	3.89–221.2	96%	56%
Biochemical *and* ultrasound	8.88	3.48–22.68	46%	96%

^1^High clinical risk: history of pancreatitis, jaundice, or cholangitis.

^2^High biochemical risk: raised ALT or ALP or bilirubin.

^3^High ultrasound risk: dilated CBD >7 mm or visualized CBD stone.

**Table 2:** Biochemical findings as separate risk factors for the presence of CBD stones.

Risk factor	Odds ratio	95% C.I.	Sensitivity	Specificity
Bilirubin	1.10	0.38–3.22	58%	83%
ALT > normal	13.7	1.57–120	96%	65%
ALP > normal	7.2	1.97–26.3	83%	82%

**Table 3:** Ultrasound findings as separate risk factors for the presence of CBD stones.

Risk factor	Odds ratio	95% C.I.	Sensitivity	Specificity
CBD > 7 mm	6.53	2.41–17.7	46%	89%
Visualised CBD stone	0.97	0.2–4.65	13%	96%
